# Molecular Detection of *blaOXA _-type_* Carbapenemase Genes and Antimicrobial Resistance Patterns among Clinical Isolates of *Acinetobacter baumannii*

**DOI:** 10.1055/s-0041-1740019

**Published:** 2022-06-13

**Authors:** Maghsoud Kafshnouchi, Marzieh Safari, Amir Khodavirdipour, Abbas Bahador, Seyed Hamid Hashemi, Mohammad Sina Alikhani, Massoud Saidijam, Mohammad Yousef Alikhani

**Affiliations:** 1 Department of Microbiology, Maragheh Islamic Azad University, Maragheh, Iran; 2 Department of Microbiology, Hamadan University of Medical Sciences, Hamadan, Iran; 3 Department of Biology, Faculty of Natural Sciences, University of Tabriz, Tabriz, Iran; 4 Department of Microbiology, Tehran University of Medical Sciences, Tehran, Iran; 5 Department of Infectious Diseases, Hamadan University of Medical Sciences, Hamadan, Iran; 6 Student Research Committee, Hamadan University of Medical Sciences, Hamadan, Iran; 7 Research Center for Molecular Medicine, Hamadan University of Medical Sciences, Hamadan, Iran

**Keywords:** *Acinetobacter baumannii*, nosocomial infection, *OXA* -type carbapenemase

## Abstract

*Acinetobacter baumannii* is a bacterium found in most places, especially in clinics and hospitals, and an important agent of nosocomial infections. The presence of class D enzymes such as OXA-type carbapenemases in *A. baumannii* is proven to have a key function in resistance to carbapenem. The aim of the current study is to determine the *blaOXA _-type_* carbapenemase genes and antimicrobial resistance among clinically isolated samples of *A. baumannii.* We assessed 100 clinically isolated specimens of *A. baumannii* from patients in intensive care units of educational hospitals of Hamadan, West of Iran. The *A. baumannii* isolates' susceptibility to antibiotics was performed employing disk diffusion method. Multiplex polymerase chain reaction was used to identify the *bla _OXA-24-like_* , *bla _OXA-23-like_* , *bla _OXA-58-like_* , and *bla _OXA-51-like_* genes. *The bla _OXA-23-like_* , *bla _OXA-24-like_*_,_ and *bla _OXA-58-like_* genes' prevalence were found to be 84, 58, and 3%, respectively. The highest coexistence of the genes was for *bla _OXA-51/23_* (84%) followed by *bla _OXA-51/24-like_* (58%). The *bla _OXA-51/23-_*_like_ pattern of genes is a sort of dominant gene in resistance in *A. baumannii* from Hamadan hospitals. The highest resistance to piperacillin (83%) and ciprofloxacin (81%) has been observed in positive isolates of *bla _OXA-23-like_* . The *A. baumannii* isolates with *bla _OXA-58-like_* genes did not show much resistance to antibiotics. Based on the results of the phylogenetic tree analysis, all isolates have shown a high degree of similarity. This study showed the high frequency of *OXA* -type carbapenemase genes among *A. baumannii* isolates from Hamadan hospitals, Iran. Thus, applying an appropriate strategy to limit the spreading of these strains and also performing new treatment regimens are necessary.

## Introduction

*Acinetobacter baumannii* is an oxidase-negative, gram-negative, nonmotile, nonfermentative coccobacilli and it is found inside most places, especially in hospitals and other health care facilities. 1 2 3 The bacterium is mostly originating from the intensive care units (ICUs). It can cause septicemia, infections of the skin, meningitis, endocarditis, urinary tract infections, soft tissues infections, and ventilator-associated pneumonia in patients in ICU wards. 4 5 6 7 Therefore, infections caused by the bacterium need early and effective antimicrobial therapy. The carbapenems are the most effective antibacterial agents in the case of clinical isolates of *A. baumannii* . 8 Nowadays, due to excessive and inappropriate use of these drugs, the resistance to carbapenems has increased. 9 *A. baumannii* can become carbapenem resistant by the acquisition of plasmid-mediated carbapenem hydrolyzing classes of A, B, and D metallo-β-lactamase enzymes. 10 Class D enzymes such as OXA-type carbapenemases, especially the intrinsic presence of *bla*_OXA-58-like_ , *bla*_OXA-24-like_ , *bla*_OXA-23-like_ , *bla*_OXA-51_ , and β-lactamases in *A. baumannii* , are proven to have a vital role in resistance to carbapenem. 11 12 13 The *OXA* -carbapenemase genes can be encoded by chromosome or plasmid and are mostly associated with insertion elements, particularly *ISAbal* . 8 With the rise of pan-drug-resistant (PDR) and multidrug-resistant (MDR) strains of *A. baumannii* all around the world, the bacterium has become a very serious threat for health care organizations. 10 14 Thus, the current study focused to discover the *blaOXA _-type_* carbapenemase genes, which, according to previous studies, contribute to more antibiotic resistance among clinical isolates of *A. baumannii.*

## Materials and Methods

### Bacterial Isolation and Identification

The cross-sectional research has been performed on 100 *A. baumannii* isolates from clinical specimens in ICU wards in educational hospitals of Hamadan, West of Iran, from December 2016 to December 2017. *A. baumannii* isolates were identified using the biochemical tests and polymerase chain reaction (PCR) targeting the carbapenemase gene of blaOXA-51-like. 15 16

#### Susceptibility Testing

The CLSI—Clinical and Laboratory Standards Institute—(CLSI, 2018) method of disk-agar diffusion 17 and interpretation by CLSI breakpoint were used to measure the susceptibility of *A. baumannii* isolates to Meropenem (10 µg), Imipenem (10 µg), Amikacin (30 µg), Gentamicin (10 µg), Ciprofloxacin (5 µg), Doxycycline (30 µg), Piperacillin (100 µg), Tobramycin (10 µg), Cefepime (30 µg), and Ampicillin/sulbactam (20 µg) antimicrobial agents. ATCC 27853- *Pseudomonas aeruginosa* was deployed in susceptibility testing as the control strain.

### Multiplex-PCR Assay

Alkaline lysis method was used for extraction of deoxyribonucleic acid (DNA). 15 The primers for PCR amplification of *bla _OXA-58-like_* , *bla _OXA-23-like_* , *bla _OXA-51-like_* , and *bla _OXA-24-like_* were used as described before ( Table 1 ). 18

**Table 1:** Primer sequences used for multiplex polymerase chain reaction amplification of bla *_OXA_* genes variants

Primer name		5′ to 3′ sequence prime	Product size (bp)	Annealing temperature (°C)	Reference
*OXA-51-like*	F	TAATGCTTTGATCGGCCTTG	353	53	Woodford (2006) 18
	R	TGGATTGCACTTCATCTTGG			
*OXA-23-like*	F	GATCGGATTGGAGAACCAGA	501	53	Woodford (2006) 18
	R	ATTTCTGACCGCATTTCCAT			
*OXA-24-like*	F	GGTTAGTTGGCCCCCTTAAA	246	53	Woodford (2006) 18
	R	AGTTGAGCGAAAAGGGGATT			
*OXA-58-like*	F	AAGTATTGGGGCTTGTGCTG	599	53	Woodford (2006) 18
	R	CCCCTCTGCGCTCTACATAC			

Multiplex-PCR was performed in a 50-µL reaction volume containing 5 µL PCR buffer 10X with MgCl _2_ , 5 µL deoxynucleotide triphosphates (2 mm), 2 µL from each reverse and forward primers (10 pmol), 0.4 µL Taq Polymerase (5U/µL), 6 µL DNA template, and 29.6 µL nuclease-free deionized water. The amplification conditions were as follows: initial denaturation at 94°C for 5 minutes followed by 30 cycles at 94°C for 30 seconds, 53°C for 40 seconds, and 72°C for 50 seconds and a final extension at 72°C for 6 minutes. Gel electrophoresis of the amplicons was performed on 1.5% agarose gel. The findings were analyzed employing a transilluminator device. Bands with 353 bp, 501 bp, 246 bp, and 599 bp were the representatives of *bla _OXA-51-like_* , *bla _OXA-23-like_* , *bla _OXA-24-like_* , and *bla _OXA-58-like_* , respectively.

### Sequencing

Three PCR products of each gene, which was separately performed, were purified and sequenced directly by Sanger dideoxy-sequencing technology in two directions using a capillary DNA analyzer (Applied Biosystems, Waltham, Massachusetts, United States).

### Phylogenetic Analysis of OXA-type Variant

The phylogenetic analysis was conducted independently for the *OXA* -type variant. The tree is drawn to scale, with branch lengths measuring the number of substitutions per site. The sequences were aligned and manually edited in consensus positions and compared with that of the sequences from GenBank. A phylogenetic tree was built by the maximum likelihood method utilizing the Tamura-Nei model with Mega software (v.7). 19

### Statistical Analysis

Statistical analysis was done using Statistical Package for the Social Sciences 23.0 (SPSS, Chicago, Illinois, United States). The Pearson chi-squared test and Fisher's exact test were used to analyze the qualitative data. All *p* -values < 0.05 were considered statistically significant.

## Results

**Fig. 1:**
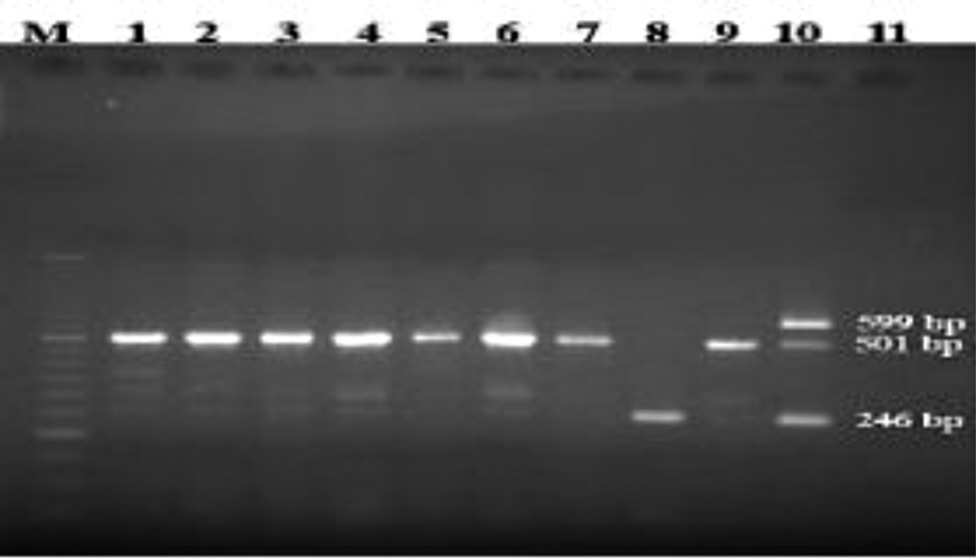
Gel electrophoresis of the blaOXA gene among the *Acinetobacter baumannii* isolates. M: 50 bp marker; 1–10: the isolates; 11: negative control. Bands with 501 bp, 246 bp, and 599 bp are the representatives of blaOXA-23-like, blaOXA-24-like, and blaOXA-58-like, respectively.

The highest resistance to piperacillin (83%) and ciprofloxacin (81%) has been observed in blaOXA23-like, and the highest resistance and sensitivity were observed for ciprofloxacin (57%) and ampicillin/sulbactam (28%) in blaOXA-24-like positive *A. baumannii* isolates, respectively. The *A. baumannii* isolates with blaOXA-58-like genes did not show much resistance to antibiotics. Considering the presence of the studied genes, 3, 58, and 84% of the 100 samples were carrying blaOXA-58-like, blaOXA-24-like, and blaOXA-23-like genes, respectively ( Fig. 1 and Table 2 ). Among the 100 *A. baumannii* isolates, 97.9% of the imipenem-resistant isolates were positive for at least one OXA-type gene. Moreover, 95.9% and 1% of the meropenem and colistin sulfate resistant isolates were carrying OXA-type genes.

**Table 2:** OXA-type patterns among carbapenem-resistant *Acinetobacter baumannii* isolates

Gene pattern	No (%)
*OXA-51*	100 (100)
*OXA-23*	84 (84)
*OXA-58*	3 (3)
*OXA-24*	58 (58)
*OXA-51/OXA-23*	84 (84)
*OXA-51/OXA-24*	58 (58)
*OXA-51/OXA-58*	3 (3)
*OXA-51/OXA-23/OXA-24*	5 (5)
*OXA-51/OXA-24/OXA-58*	2 (2)
*OXA-51/OXA-23/OXA-24/OXA-58*	1 (1)

### Results of Phylogenetic Tree Analysis of OXA-type Variant

**Fig. 2:**
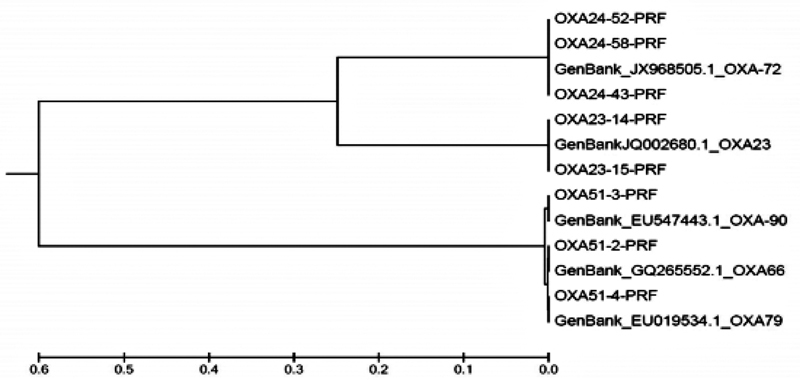
Phylogenetic tree built based on the sequence isolates by maximum likelihood method using the Tamura-Nei model. Based on the results, all isolates have shown a high degree (95–100%) of similarity.

The sequences received from the isolates were confirmed as the *OXA* -type variants and were deposited in the GenBank under the accession numbers as follows: *bla _OXA-51-like_* : KJ451411, KJ451412, and KJ451413; *bla _OXA-23-like_* : KJ451414 and KJ451415; and *bla _OXA-24-like_* : KJ451416, KJ451417, and KJ451418 ( Fig. 2 ). Analysis of the phylogenic tree demonstrates that all isolates have shown a high degree of similarity.

### Correlation between Contributions of bla _OXA_ Genes with Antibiotic Resistance

The relationship between blaOXA-types and resistance to different antibiotics was determined by statistical analysis. The prevalence of oxacillinases enzymes encoding genes in antibiotic-resistant isolates was higher than the susceptible strains, but there was no statistically significant relationship ( Table 3 ).

**Table 3:** Statistical analysis of the correlation between the presence of bla _OXA_ -type genes and antibiotic resistance in *Acinetobacter baumannii* isolates

Gene		OXA23		Fisher exact test	Exact sig.(two-sided)	OXA24		Fisher exact test	Exact sig.(two-sided)	OXA58		Fisher exact test	Exact sig.(two-sided)
Antibiotic		Positive	Negative			Positive	Negative			Positive	Negative		
Meropenem	R	79%	14%	0.671	0.692	56%	37%	1.855	0.374	3%	90%	1.608	1
	I	2%	0			2%	0%			0%	2%		
	S	4%	1%			2%	3%			0%	5%		
Imipenem	R	73%	12%	2.37	0.429	52%	33%	2.066	0.351	2%	83%	2.799	0.389
	I	1%	1%			2%	0%			0%	2%		
	S	11%	2%			6%	7%			1%	12%		
Amikacin	R	72%	12%	1.422	0.443	51%	33%	1.099	0.703	2%	82%	3.212	0.217
	I	8%	1%			6%	3%			0%	9%		
	S	5%	2%			3%	4%			1%	6%		
Gentamicin	R	74%	13%	2.33	0.324	54%	23%	3.58	0.116	3%	84%	1.29	1
	I	1%	1%			2%	0%			0%	2%		
	S	10%	1%			4%	7%			1%	11%		
Ciprofloxacin	R	81%	14%	1.37	0.549	57%	38%	1.75	0.580	3%	92%	2.374	1
	I	1%	0%			0%	1%			0%	1%		
	S	4%	1%			3%	1%			0%	4%		
Doxycycline	R	24%	5%	2.45	0.320	18%	11%	0.287	0.902	1%	28%	2.092	0.387
	I	34%	3%			21%	16%			0%	37%		
	S	27%	7%			21%	13%			2%	32%		
Piperacillin	R	83%	15%	1.117	1	59%	39%	2.033	0.642	3%	95%	3.541	1
	I	1%	0%			1%	0%			0%	1%		
	S	1%	0%			0%	1%			0%	1%		
Tobramycin	R	65%	11%	1.386	0.575	47%	29%	0.824	0.676	3%	73%	1.19	1
	I	2%	1%			2%	1%			0%	3%		
	S	18%	3%			11%	10%			0%	21%		
Cefepime	R	79%	13%	2.089	0.443	55%	37%	0.501	1	3%	89%	1.50	1
	I	4%	2%			4%	2%			0%	6%		
	S	2%	0%			1%	1%			0%	2%		
Amp/sul	R	30%	3%	2.94	0.243	21%	12%	1.20	0.561	0%	33%	3.098	0.155
	I	20%	2%			11%	11%			2%	20%		
	S	25%	10%			28%	17%			1%	44%		

Abbreviations: Amp/sul, ampicillin/sulbactam; I, intermediate; R, resistant; S, sensitive; sig., significance.

## Discussion

Currently, carbapenem resistance in *A. baumannii* isolates has become a worldwide problem. This study's results demonstrated that significant numbers of clinical isolates of *A. baumannii* , especially resistant to imipenem and meropenem isolates, were affirmative for the variety of *OXA* gene expressions. Commonly, the carbapenem antibiotics are highly active against *A. baumannii* isolates and resistant to β-lactamase enzymes. However, the resistance to these compounds is a problematic issue and is not a result of the presence of a distinct mechanism, while a combination of diverse mechanisms such as enzymatic and nonenzymatic ones are involved. The most significant mechanism of resistance to carbapenems is the enzymatic hydrolysis, arbitrated by the carbapenemases. 11 20 Recently, the number of β-lactamase enzymes in class D has increased significantly. Some of the *OXA* -type carbapenemases are widely found in *A. baumannii* isolates. However, many of the *OXA* -type carbapenemases illustrate simply weak catalytic activity, but resistance to carbapenems may be caused by a combined action of different *OXA* -type carbapenemase. 21 Feizabadi et al in 2008 studied the susceptibility to antimicrobial and *blaOXA* genes distribution among *Acinetobacter* spp. in Tehran hospitals. They reported the coexistence of *bla _OXA-51/23_* and *bla _OXA-51/24-like_* in 25% ( *n* = 32) and 17.9% ( *n* = 23) of their studied isolates, respectively. Over 70% of their studied carbapenem-resistant isolates had at least two genes encoding OXA-type carbapenemase. 22 In our study, the highest coexistence was for *bla _OXA-51/23_* (84%) followed by *bla _OXA-51/24-like_* (58%), which is considerably higher than their report from Tehran and indicates an elevation in the prevalence of *A. baumannii* isolates carrying these genes over the time. In the current research, comparable to another research performed in Iran, the most commonly found OXA-type carbapenemase was OXA-23 among other carbapenem-resistant isolates, next to OXA-24. 23 24 Zafari et al reported a high prevalence of OXA-type carbapenemases among *A. baumannii* isolates in Tehran in 2017. They studied *bla _OXA-51-like_* , *bla _OXA_*_ - *58-like*_ , *bla _OXA-23-like_* , and *bla _OXA-24-like_* genes among 100 isolates of carbapenem-resistant *A. baumannii* . All of their studied carbapenem-resistant *A. baumannii* isolates were MDR and extensively drug-resistant and even 2% of the isolates were PDR. The *bla _OXA-24-like_* and *bla _OXA-23-like_* prevalence genes were reported to be 22% and 81%, respectively. They concluded that there are very few therapeutic options for the treatment of carbapenem-resistant *A. baumannii* infections. 23 In the present study, just similar to the findings of Zafari et al in Tehran, 84, 58, and 3% of the *A. baumannii* isolates from Hamadan, west of Iran, were carrying *bla _OXA-23-like_* , *bla _OXA-24-like_* , and *bla _OXA-58-like_* genes, respectively. The very high prevalence of drug resistance among *A. baumannii* isolates was reported in Hamadan in 2013. 24 Bardbari et al investigated the *OXA* -type gene presence including *bla _OXA-58_* and *bla _OXA-23_* , *blaOXA _-24_* among 75 clinical and 32 environmental strains of the *A. baumannii isolates* in Hamadan in 2017. Their results showed that 80.4, 30.8, 0, and 30.8% of their studied *A. baumannii* isolates have *bla _OXA-23/24_* , *blaOXA _-24_* , *bla _OXA-23_* , and *bla _OXA-58_* genes. 25 Their results are similar to the finding of the present study. Given the fact that both studies are conducted in the same area but on different isolates, the similar results can be explained. In a study that was prepared by Zowawi et al, OXA-23 was identified as the highest type (91.5%) of carbapenemases in carbapenem-resistant *A. baumannii* isolates from hospitals of Bahrain, Saudi Arabia, Oman, Kuwait, United Arab Emirates, and Qatar followed by OXA-24 (4.3%). 26 Also, research works from other countries have reported that OXA-23 as a dominant type in carbapenem-resistant isolates has been accompanying outbreaks. 27 28 Similar to our results, some studies have reported the low prevalence of OXA-58 among carbapenem-resistant isolates from the east (0.5%), and northwest of Iran (3.2%). 26 29 The outcome of our research confirmed the high prevalence of OXA-type carbapenemase between carbapenem antibiotic-resistant *A. baumannii* bacteria; furthermore, correct prescription of antibiotics and effective infection control monitors for preventing the extent of resistant isolates are required.

## Conclusion

This study's results demonstrated that carbapenem resistance is increasing among *A. baumannii* clinical isolates in our area and is often associated with multidrug resistance. Moreover, the diversity of *bla* OXA genes was high among these isolates and OXA-23 is the most important carbapenemase mechanism responsible for the resistance phenotype.
